# Portable sequencing, genomic data, and scale in global emerging infectious disease surveillance

**DOI:** 10.1002/geo2.66

**Published:** 2018-12-01

**Authors:** Liam P. Shaw, Nicola C. Sugden

**Affiliations:** ^1^ UCL Genetics Institute UCL London UK; ^2^ Nuffield Department of Medicine University of Oxford Oxford UK; ^3^ Centre for the History of Science Technology and Medicine University of Manchester Manchester UK

**Keywords:** emerging infectious disease surveillance, global health, human microbiome, more‐than‐human geography, portable sequencing

## Abstract

Emerging infectious diseases (EIDs) occur when pathogens unpredictably spread into new contexts. EID surveillance systems seek to rapidly identify EID outbreaks to contain spread and improve public health outcomes. Sequencing data has historically not been integrated into real‐time responses, but portable DNA sequencing technology has prompted optimism among epidemiologists. Specifically, attention has focused on the goal of a “sequencing singularity”: the integration of portable sequencers in a worldwide event‐based surveillance network with other digital data (Gardy & Loman, *Nature Reviews Genetics, 19*, 2018, p. 9). The sequencing singularity vision is a powerful socio‐technical imaginary, shaping the discourse around the future of portable sequencing. Ethical and practical issues are bound by the vision in two ways: they are framed only as obstacles, and they are formulated only at the scales made visible by its implicit geography. This geography privileges two extremes of scale – the genomic and the global – and leaves intermediate scales comparatively unmapped. We explore how widespread portable sequencing could challenge this geography. Portable sequencers put the ability to produce genomic data in the hands of the individual. The explicit assertion of rights over data may therefore become a matter disputed more at an interpersonal scale than an international one. Portable sequencers also promise ubiquitous, indiscriminate sequencing of the total metagenomic content of samples, raising the question of what (or who) is under surveillance and inviting consideration of the human microbiome and more‐than‐human geographies. We call into question a conception of a globally integrated stream of sequencing data as composed mostly of “noise,” within which signals of pathogen “emergence” are “hidden,” considering it instead from the perspective of recent work into more‐than‐human geographies. Our work highlights a practical need for researchers to consider both the alternative possibilities they foreclose as well as the exciting opportunities they move towards when they deploy their visions of the future.

## Abstract 2

The anticipated ubiquity of portable sequencers will have important implications for the future of emerging infectious disease (EID) surveillance. We argue that this future is being shaped in part by a powerful socio‐technical imaginary, the “sequencing singularity” vision, which contains an implicit geography that privileges the international scale at the expense of others. This article challenges that geography by considering some of the ethical and practical concerns, as well as opportunities, that could arise at other scales made visible by widespread in situ sequencing.

e00066

## 1 INTRODUCTION

Emerging infectious diseases (EIDs) occur when microbial pathogens spread into new contexts. EIDs are unpredictable, can spread quickly, and often have devastating consequences. High‐profile examples of EIDs include HIV/AIDS (Gao et al., 1999), Ebola virus (Carroll et al., 2015) and Zika virus (Faria, Azevedo, et al., 2016). Recent outbreaks of Ebola and Zika have caused tens of thousands of deaths and prompted global panic, further galvanising infectious disease researchers’ sense of urgency and commitment to developing approaches to monitor and manage EIDs. This work includes an interest in realising the potential of new technologies and big data to minimise the consequences of future outbreaks through initiatives such as the continued development of infectious disease surveillance systems managed by governments, international bodies and academic consortia. Infectious disease surveillance systems are designed to meet the temporal challenge of fast‐spreading EIDs by rapidly identifying outbreaks of new pathogens. Their goal is to inform earlier and better interventions in order to contain spread and improve public health outcomes.

A great deal of recent work in the surveillance of infectious disease has called for the integration of different surveillance networks into a unified global system (Barboza, 2017; Brookes et al., 2015; Gardy & Loman, 2018; Gardy et al., 2015; Moon et al., 2015). Just as infectious diseases have no regard for national borders, the argument goes, nor should attitudes toward data sharing: global risk must be met by global surveillance efforts across political boundaries to prevent loss of life.^1^ By facilitating fast global responses, EID surveillance is deemed to have the potential to address the geographical as well as the temporal challenge presented by EIDs. In this article, we discuss some of the questions that could arise from the integration of genomic data into a global surveillance system.

Genomic sequencing is useful in EID surveillance because the genomes of pathogens can be used to elucidate the development of an outbreak. Earlier techniques involved targeting specific regions of the pathogen genome in so‐called “molecular epidemiology”; in “genomic epidemiology” the entire genome is sequenced, providing much more information (Struelens & Brisse, 2013). Early on in outbreaks, information from sequencing can be used to estimate the evolutionary rate of the pathogen (e.g., the influenza A H1N1 pandemic of 2009: Fraser et al., 2009). Mutations in the genome can then be used to reconstruct a phylogeny, where differences between sampled strains due to their evolutionary history allow the reconstruction of their transmission and origins. Genomic epidemiology can therefore assist in the identification of transmission mechanisms to inform public health responses from national governments and non‐governmental organisations (NGOs). For example, a pathogen which has emerged from a single zoonosis into humans and spreads via human‐to‐human transmission (e.g., the West African Ebola outbreak of 2013–2016: Tong et al., 2015) will have a very different phylogeny to one which emerges from repeated introductions into the human population by animal‐to‐human transmission (e.g., sporadic *Mycobacterium bovis* infections in the UK between 2005 and 2010: Stone et al., 2012). Differentiating between transmission routes enables better informed public health responses, including more specific containment advice for the public: for example, evidence from genomic data that a particular strain is transmitted from animals to humans can be used to explain and justify control measures in agricultural settings (e.g., swine‐to‐human transmission of influenza A H3N2 in Ohio, USA in 2012: Bowman et al., 2014). Genomic epidemiology has shown in some settings that conventional epidemiology fails to capture complex transmission routes (e.g., hospital cases of *Enterococcus faecium* in the UK: Raven et al., 2017), suggesting that infection control procedures based on conventional information are inadequate. Genomic sequencing can also be used to retrospectively identify “cryptic” transmission, where pathogens have circulated prior to the first known case (e.g., the pre‐detection circulation of Zika virus in Brazil: Faria et al., 2017). Historically, genomic data for EID pathogens has not been widely available to researchers for the purposes of surveillance, or during ongoing outbreaks, although it can still be used in attempts to better understand previous outbreaks and identify their origins (e.g., the origin of HIV‐1 in chimpanzees: Gao et al., 1999).

One reason for the exclusively retrospective nature of genomic EID surveillance is that sequencing machines (henceforth, “sequencers”) have heretofore been large, slow and expensive. The ability to sequence has been (generally speaking) restricted to organisations with significant resources at their disposal – whereas EIDs themselves disproportionately affect some of the least wealthy areas of the world. Frequently, samples are taken from low‐income countries during EID outbreaks to high‐income countries to be sequenced. This movement and its consequences are often characterised by a highly unequal distribution of rights, responsibilities, access, and knowledge. Significant conflicts have emerged from the asymmetric relationships arising in the collection, ownership, and use of data derived from such samples. Examples include the conflicting priorities of the US government in influenza vaccine development requiring the patenting of viral sequences (Hammond, 2008, pp. 22–23); researchers’ complaints of the “bureaucratic nightmare” of shipping samples potentially containing Ebola virus (Kupferschmidt, 2017); and the dispute between the Saudia Arabian government and Dutch researchers over a novel coronavirus (Butler, 2013; MacKenzie, 2013; – discussed in more detail in Section 4). Despite increasing demand for protections for the “owners” of samples (WHO, 2017), normal research protocols are not necessarily followed during rapid outbreaks; during the 2013–2016 West African Ebola outbreak, the World Health Organization Ethics Research Committee (WHO‐ERC) decided to approve studies using such samples “based on researcher commitment to put appropriate agreements/processes in place” for sample and data ownership “[i]n view of the urgency” of the situation (Alirol et al., 2017, p. 7).

Increased data sharing across disease surveillance systems is – in principle – endorsed by many governments, funders and pharmaceutical companies, for example the UK government (O'Neill, 2016, p. 32), the Wellcome Trust (2018), and the International Federation of Pharmaceutical Manufacturers & Associations (IFPMA, 2016, 2018). This attitude has led to the creation of collaborative endeavours such as the Infectious Diseases Data Observatory (IDDO, 2018), which aims to link the “global infectious disease community across the research and humanitarian sectors” to share data on EIDs. Some have criticised data sharing as usually having a hidden agenda (Mirowski, 2018) and even proponents acknowledge that ensuring the equitability of data sharing is challenging (Edelstein et al., 2018). Concurrently, over the past five years significantly smaller, faster and less expensive sequencers have been developed to the point where sequencers are now portable (definitions of “portable” vary, but we assume that a “portable sequencer” is one that can be easily transported and used by a single person in the field). Portable sequencing technologies allow the processing of samples in situ. For example, the Zika in Brazil Real‐time Analysis (ZiBRA) project recently created a portable genome sequencing laboratory in a mobile trailer by using Oxford Nanopore MinION sequencers (Faria, Sabino, et al., 2016). This project was a primarily UK–Brazil collaboration involving public health researchers in Brazil and a global community of researchers, and had a stated “open data release policy” to release sequencing datasets “as soon as we produce them” (Zibra Project, 2016). The ZiBRA project has been referenced as an impressive proof of principle that portable sequencers in conjunction with this type of data sharing can be used effectively and productively in outbreak settings (Leggett & Clark, 2017; Loose, 2017)**.**

Researchers in microbial genomics are now advocating for the systematic inclusion of genomic data in EID surveillance. In a recent article, Gardy and Loman (2018) reviewed the state of the art and made recommendations for future developments. Both have considerable experience in the synthesis of genomic data for outbreak responses. For example, Loman was involved in the crowd‐sourced analysis of a *E. coli* O104:H4 outbreak in May/June 2011 in Germany (Rohde et al., 2011), and Gardy has used genomic sequencing in conjunction with social network analysis to elucidate chains of transmission in tuberculosis (Gardy et al., 2011). In their review, Gardy and Loman outline the possibility of linking portable sequencers via a worldwide network to achieve a “sequencing singularity”: “the moment at which pathogen, environmental and digital data streams are integrated into a global surveillance system” (Gardy & Loman, 2018, p. 18).^2^ This statement is emblematic of a broad vision for the future of EID surveillance, one that extrapolates from the increasing tendency for data aggregation from multiple sources to a future “singularity”: a state of affairs whereby omniscience in the form of real‐time and expansive surveillance could facilitate near omnipotence in responses to EIDs. In words attributed to the physician Larry Brilliant: “Outbreaks are inevitable; epidemics are not” (Loman, 2018).

Hypotheses of technological singularity have many possible interpretations (Eden et al., 2012) but the use of the term by Gardy and Loman invites comparisons with “The Singularity,” the hypothetical point where artificial intelligence leads to runaway technological growth (see Kurzweil, 2005). A singularity is a point of no return, beyond which further developments and consequences are unforeseeable; hence Hanson's (2008, p. 45) general definition of a singularity as “an overwhelming departure from prior trends, with uneven and dizzyingly rapid change thereafter.” The “sequencing singularity” vision is indisputably one with grand ambitions, matched by a name that connotes dramatic, epoch‐changing technological change. If we are, indeed, on the brink of such sweeping change, it is necessary to raise some of the other possible consequences as a matter of concern.

Infectious disease researchers, including Gardy and Loman, do recognise that the idea of widespread use of portable sequencing for EID surveillance poses some ethical problems. However, we believe the concerns that have been raised thus far are typically bound by the “sequencing singularity” vision in two ways: they are framed only as obstacles to the realisation of the vision, and they are formulated only at the scales recognised by its implicit geography – a geography prescribed by the dominant contemporary strategies and aesthetics of EID surveillance and response. With specific reference to the promises of portable genomic sequencing, we wish to disrupt that geography in this article.

Our interest in this topic comes from our different disciplinary perspectives: computational biology applied to the microbiome (Liam Shaw, [L.S]) and the history of science, technology and medicine (Nicola Sudgen, [N.S]). Our combined expertise covers the technical practice of managing and analysing genomic data (L.S.) as well as the historical, sociological and philosophical analysis of science (N.S.). Our informal discussions about genomic sequencing crystallised in light of recent events related to digital data (see Authors’ Note at the end), and motivated this article. Discussing EID surveillance across disciplines required both of us to engage with unfamiliar material. While we have endeavoured to draw on and acknowledge relevant existing work, we remain aware that omissions are inevitable, and that it has been necessary to pass over some areas almost cursorily. We hope that such shortcomings may be taken as a mark of the wealth and diversity of pertinent material rather than a comment on the quality or significance of specific research areas, and that our contribution promotes a way of thinking rather than any final pronouncement.

We begin by accounting for current enthusiasm for portable sequencing, urging that this excitement be tempered, before moving to understand the “sequencing singularity” as a socio‐technical imaginary with an implicit worldview which incorporates a specific geography (and much more besides). We then discuss the relationship between visualisation in contemporary EID surveillance and genomics and the “sequencing singularity” vision, before challenging the vision's geography by exploring two possible consequences of ubiquitous portable sequencing. First, we demonstrate that widespread use of portable sequencing in EID surveillance could result in the emergence of ethical and practical problems which require the recasting of questions of biological sovereignty at an interpersonal rather than international level. Second, we suggest that the “sequencing singularity” vision privileges a particular understanding of what constitutes “signal” and “noise” within metagenomic datasets, foreclosing other exciting possibilities. Ultimately, the widespread use of portable sequencers for the purpose of EID surveillance will have consequences beyond those envisaged and promoted within the “sequencing singularity” vision and at scales occluded by its implicit geography. We therefore urge researchers to be sensitive to the possibilities foreclosed by the privileging of certain scales at the expense of others.

## 2 IMAGINING THE SEQUENCING SINGULARITY

Outbreaks of EIDs are typically initiated by chance events, and are therefore inherently unpredictable. The West African Ebola virus epidemic of 2013–2016 is believed to have originated from the infection of two‐year‐old Emile Ouamouno in Guinea. After his death, the virus went on to infect over 28,000 other people, killing at least 11,000 (WHO, 2016). The unpredictability and devastating consequences of EIDs have been compared to those of gigantic forest fires started from a single spark, with the international aid efforts that start only when the outbreak is already underway likened to “valiant bucket brigades organized after the fire is out of control” (Worobey, 2017, p. 356). Worobey and others have stressed the need for constant and extensive surveillance to detect unfolding outbreaks and enable international organisations to intervene as quickly as possible. The fundamental unpredictability of such events means that the dominant method of relying on targeted surveillance narrowly focused on specific sources risks failure to detect outbreaks emerging from other sources. A perception that the quantity or quality of data available is inadequate can also render judgements with important political consequences more controversial, such as the categorisation of an ongoing incident as a pandemic (Abeysinghe, 2013) or the predicted number of future cases. The latter is a highly politicised figure that frequently dominates the discourse around EID response, but is often based on assumptions about the poor quality of available data. Epidemiologists attempt to account for suspected underreporting of initial cases, which often leads to what one might call “conservative overestimations.” For example, the Centers for Disease Control and Prevention's September 2014 prediction for the number of Ebola virus disease cases in Liberia and Sierra Leone by January 2015 in the absence of any intervention was nearly tripled from 550,000 to 1.4 million “when corrected for underreporting” (Meltzer et al., 2014, p. 3), a number that was repeated in global media and framed as a “set of ominous projections” of a “worst‐case scenario” (Grady, 2014). Invoking the unknown is an important strategy for scientists acting as “prophets,” who aim to be “recognized as reasonable and accepted as authoritative” (Caduff, 2014, p. 296). MacPhail (2014, p. 136) has demonstrated how this sort of dramatic uncertainty, referenced by scientists as “the absence of ‘good data,’” is often deployed strategically to strengthen scientific authority. It is also frequently used as a key argument in the drive to incorporate more and more data into surveillance systems (see e.g., Barboza, 2017).

Surveillance is a broad and contested concept. For the purposes of this article, we take “surveillance” to mean “the organized monitoring of the activities of actors” for the purpose of governing (Henry, 2009, p. 95). We use “EID surveillance” as a catch‐all term referring to monitoring activities that are intended to alert authorities to the presence of outbreaks and to inform their responses. EID surveillance itself takes many forms, and it is not our aim to provide an extensive taxonomy here. We do, however, wish to highlight a current trend away from traditional indicator‐based surveillance. Indicator‐based approaches specify known cut‐offs for outbreaks based on regularly updated indicators from laboratories or healthcare facilities. In contrast, event‐based surveillance systems are based on “unstructured descriptions and reports” which are then rapidly assessed for the risk posed to public health (WHO, 2008, p. 3). The emphasis is on quickly detecting the “signal” of an event that poses a risk to public health from the “noise” of other events. The spectrum of data envisaged for inclusion in event‐based surveillance is much wider than that used in indicator‐based surveillance, with any digital information that can be scraped from Internet sites being utilised. The drive towards increasingly powerful event‐based surveillance has already led to the development of multiple approaches: a recent review identified 50 such event‐based Internet systems (O'Shea, 2017). This proliferation in turn has led to many to call for initiatives in the direction of a “super‐system … to pool expert systems’ expertise” (Barboza et al., 2013, p. 8). WHO plan to roll out such a “super‐system” in 2018, integrating traditional and social media with government sources and processing over one million media articles a week (Barboza, 2017, p. 11).

This article is centred on the inclusion of sequencing data into the burgeoning world of digital event‐based surveillance systems. Due to the physical size of sequencers, sequencing data has until recently been physically tied to established laboratories and in turn restricted to informing indicator‐based systems. Recent optimism about portable sequencers arises from their potential to liberate sequencing data from such limitations and ultimately enable an instantly globally accessible digital sequencing data stream. Presently, such hopes are intimately linked to devices like the Oxford Nanopore MinION: a palm‐sized sequencer which offers direct DNA or RNA sequencing in “REAL Real‐time [sic]” (Oxford Nanopore, 2018). Portable sequencers like the MinION are intended “to enable the analysis of any living thing, by any person, in any environment” (Oxford Nanopore, 2018). In the context of EID surveillance, indiscriminate sequencing of the total genomic content of samples without a species bias (metagenomic sequencing) would enable an “event‐based” approach to pathogen detection. Although there are still considerable technological obstacles to the deployment of widespread portable sequencing, the MinION is emblematic of the ostensible benefits of portable sequencing, particularly with regard to its incorporation into global digital networks in the “sequencing singularity.”

It should not be surprising that genomic epidemiologists are enthusiastic about the prospect of a global surveillance network. Digital communication networks are familiar and important tools in their day‐to‐day work, as is an embrace of the new and a playful exploratory attitude towards technology and data. For example, Gardy (2017) understands her first paper using whole genome sequencing to characterise multiple person‐to‐person transmission as coming from “messing around with data.” Clive Brown, Oxford Nanopore's CTO, foresees a future where “sequencers will become like telescopes: a formerly boutique scientific instrument that you can now buy from a toy store” (Yong, 2016). Yet the data produced by sequencers is profoundly personal and intimately entangled with our lives, bodies and selves. Much is therefore at stake as this technology develops.

Considering the implications of portable sequencers in EID surveillance requires careful thought about what happens when a technology becomes ubiquitous. We urge that enthusiasm for the opportunities afforded by portable sequencing be tempered by an appreciation of the multivalence of technology. On this point, we think that a historical example is instructive. From the early 1980s, early adopters of the Internet used the discussions system Usenet to communicate with each other. Many of these early adopters shared a cyber‐utopian belief in “the emancipatory nature of online communication,” which offered particular transformational potential for society (Morozov, 2012, p. xiii). But when AOL offered Usenet access to its customers in September 1993, the system was flooded with new users who did not subscribe to the same vision and values, and whose participation saw the platform evolve in other, unexpected, directions. Some previous users felt this was responsible for a change and “inexorable decline” in the quality of Usenet discussions, an “Eternal September” (Raymond, 2003).^3^ The technology also became increasingly corporatised and controlled by monopolies, which had corresponding effects on the “open Internet spirit” (Krämer et al., 2013, p. 795). For example, many early Internet enthusiasts supported strict net neutrality, which “prohibits Internet service providers from speeding up, slowing down or blocking Internet traffic based on its source, ownership or destination” (Krämer et al., 2013, p. 796). During the 1990s the Internet service provider AOL was the leading corporate campaigner for a US national policy on net neutrality (Chiappinelli et al., 2007, p. 960). However, after AOL merged with the media company Time Warner in 2000 (one of the largest mergers in US history) it had a vested interest in operating a network with “favorable treatment” for its own content, and therefore ceased campaigning for net neutrality (Chiappinelli et al., 2007, pp. 960–961). Corporate dynamics therefore shape the flow of information through networks. Our point in drawing a parallel between early Internet users and contemporary portable sequencing enthusiasts is to suggest that the latter, like the former, risk committing what we tentatively term the “Early Adopter Fallacy”: the belief by a community of early adopters of a technology that their shared vision and values are straightforwardly related to the technology itself, and will therefore naturally continue to be a fundamental element of its use. However, the expansion in use of a technology is a process in which technology, users, corporations and values continually recombine in unexpected ways. From design and production to deployment and beyond, technologies are fraught with politics, entangled in complex and changing relations with people, things and places. Ultimately, technologies almost always surprise us in some way as they evolve; this will undoubtedly be true for portable sequencers. This is certainly not an inherently bad thing, but it is incumbent upon us to do our best to try to recognise and attend to the problems – and indeed opportunities – presented by such under‐determination.

Proponents of portable sequencers foresee opportunities for their application in diverse settings where the flow of information they can produce could be of value. Clive Brown has said that “the wealth of information we can *intercept* [with portable sequencers] can change the way people live” (Clark, 2015; our emphasis). Such language reinforces a conception of data as the manifestation of a constant flow of information that is circulating in the external world, streaming away and becoming useless unless we intervene and capture it for (and with) our own devices. Within the “sequencing singularity,” the aim is to intercept and transform sufficient information related to EIDs to reliably map the relevant parts of the “real” world in “real time.” Gardy and Loman (2018, p. 16) imagine that in the near future novel EID events will be routinely detected with the help of portable sequencers:

It is 2027, and our planet's changing climate and land‐use patterns have meant that new emerging infectious diseases (EIDs) are spilling over into humans from wildlife reservoirs with increasing frequency … a global public health consortium has implemented an online surveillance tool that scans the digital output of citizens, news organisations and governments in those regions, including data from local retailers on key health‐related products…In one such region, the syndromic surveillance system reports higher‐than‐average sales of a common medication used to relieve fever. Spatial analysis of the data from the pharmacies in the region suggests that the trend is unique to a particular district; a follow‐up geographic information system (GIS) analysis using satellite data reveals that this area borders a forest and is increasingly being used for the commercial production of bat guano. An alert is triggered, and the field response team meets with citizens in the area. Nasopharyngeal swabs are taken from humans and livestock with fever as well as from guano and bat tissue collected in the area. The samples are immediately analysed using a portable DNA sequencer coupled to a smartphone. An app on the phone reports the clinical metagenomic results in real time, revealing that … a novel coronavirus makes up the bulk of the microbial nucleic acid fraction. The sequencing data are immediately uploaded to a public repository as they are generated, tagged with metadata about the host, sample type and location and stored according to a pathogen surveillance ontology. The data release triggers an announcement via social media of a novel sequence, and within minutes, interested virologists have created a shared online workspace and open lab notebook to collect their analyses of the new pathogen.

We take this as a paradigmatic statement of the “sequencing singularity.” The vision seems to contain a series of explicit and implicit assumptions, including that:

1. The creation of such a system is a moral imperative due to increasing frequency of EIDs.
2. The system is overseen by a benevolent consortium of actors.
3. Individuals freely consent to give their data to this consortium.
4. Social media and the Internet play a positive role in the dissemination of information.
5. Data upload is tied to location, but data download and access is global.

These are, we argue, some of the defining elements of a particular “socio‐technical imaginary” associated with the “sequencing singularity”: we call it the “sequencing singularity” vision. Socio‐technical imaginaries are “collectively held, institutionally stabilised, and publicly performed visions of desirable futures, animated by shared understandings of forms of social life and social order attainable through, and supportive of, advances in science and technology” (Jasanoff, 2015). A socio‐technical imaginary operates as “neither cause nor effect in a conventional sense but rather a continually articulated awareness of order in social life … and a resulting commitment to that order's coherence and continuity” (Jasanoff, 2015, p. 26). Previous work has explored the socio‐technical imaginary that underlies the “strategic framework” of “global health security” (Lakoff, 2015, p. 300), and we see the “sequencing singularity” vision as related to but separate from this imaginary, centred as it is on a specific technology. As a socio‐technical imaginary, the “sequencing singularity” vision has predictive, persuasive and productive power; it is active in shaping both the research landscape we operate within and our understanding of it. In publicly performing the “sequencing singularity” vision, Gardy and Loman (2018) reinforce its attendant morality, politics, and – as we explore here – geography.

Understanding the “sequencing singularity” vision as a socio‐technical imaginary, we attempt to take some steps towards recognising, and taking seriously, both the enmeshment of portable sequencing technology with people and things, and the significance of the vision's adherents’ ongoing roles in enacting a particular state of affairs and way of living. In this article we open a dialogue with this imaginary, with a view to highlighting other possible states of affairs to those already envisioned. By exploring the impact of portable sequencers on the visibility of certain previously neglected intermediate scales, we try to articulate some of these troubling and promising alternatives.

We choose to investigate the geography of the “sequencing singularity” vision through three lenses: imagery, ownership, and biological complexity. First, what is the contemporary aesthetic of EID surveillance, and how does this relate to the geography of the “sequencing singularity” vision? We examine current visualisations of genomic data in EID surveillance, which connect the genomic and the global but occlude intermediate scales. Second, we look at the ownership of genomic data. We argue that norms of ownership are still heavily influenced by the historical development of sequencers and circumstances of their use, and that portable sequencers will disrupt these norms by virtue of their reduced size. In other words, we feel the “sequencing singularity” vision does not take sufficient account of the unsustainability of existing practices around data ownership. Finally, we explore the prospect of untargeted metagenomic sequencing producing vast quantities of data that reflect the biological complexity of more‐than‐human geographies. Metagenomic data is figured as predominantly “contamination” or “noise” by those interested in EID surveillance, but within other contexts of inquiry (see Leonelli, 2016) it undoubtedly contains valuable information which could be exploited for various purposes.

## 3 SEEING EIDS

The sequencing singularity vision has an implicit geography imported from the dominant strategies and aesthetics of contemporary EID detection and response. Data in EID surveillance must be rendered visible (both literally and metaphorically) in order to be used. Contemporary visualisations of EID surveillance and genomic data are both functional and symbolic: they facilitate use of the data, and they also represent a strategy for knowing and interacting with EIDs. These visualisations make visible certain scales and occlude others.

Previous work has highlighted the different discourses that can be associated with “global” images. For example, Cosgrove analysed the Apollo space photographs of the entire globe and identified two distinct but related readings of the same image (Cosgrove, 1994), and has explored the role of visual knowledge more generally in geography (Cosgrove, 2008). The role of visualisations of the global is an active topic of research (see e.g., the recent *Geo* special issue on “Visualising the global environmental: new research directions”; Grevsmühl, 2017). Here, we focus in on the global imagery used by one flagship visualisation project in EID surveillance.

Nextstrain (nextstrain.org) is an open‐source project that uses interactive data visualisations to make pathogen genomes visible to virologists, epidemiologists, public health officials and citizen scientists. The project's philosophy is to implement “robust bioinformatic pipelines to synthesize data from across research groups,” which its creators believe offers “the best capacity to make epidemiologically actionable inferences” (Bedford et al., 2018). Nextstrain is a compelling example of the enthusiasm for and celebration of “open science”; in 2017 it was awarded the first Open Science Prize (2017). Nextstrain's underlying data visualisation framework is called “auspice” – in the words of its developers, “a prophetic sign” (Bedford & Neher, 2018). Unlike static maps, auspice produces malleable and flexible maps that are viewed in the user's browser. These visualisations powerfully contribute to a “real‐time” feeling of dynamism and change on a global scale, and stand in stark contrast to the static figures presented in scientific publications (including, inevitably, our examples below).

**Figure 1:**
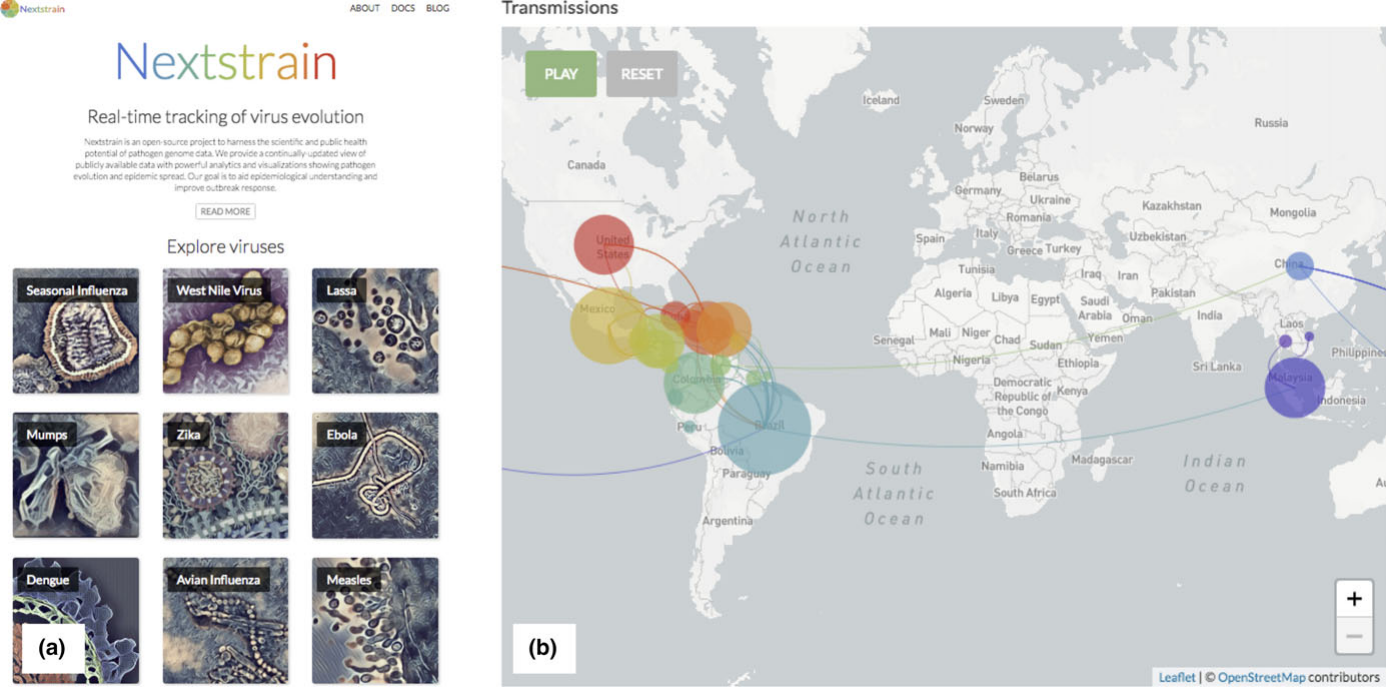
Seeing emerging infectious diseases with nextstrain. (a) The nextstrain homepage offers the opportunity to “Explore viruses” through images of their microscopic appearances. (b) A visualisation of the reconstructed transmission of Zika virus around the globe, which can be played by the user in real‐time. (Both images screenshotted from nexstrain.org on 10/07/2018, authors’ own work).

Nextstrain's global maps are especially compelling, even to the layperson. Coloured circles hover and pulse ominously over infected areas, and connecting lines show routes of transmission webbing across the globe, producing a sense of international connectedness and moving danger (Figure 1). The endangered globe is a defining topology of EID surveillance, which is concerned with a movement of pathogens around that globe, and therefore must itself oscillate between the global and microscopic scales.

**Figure 2:**
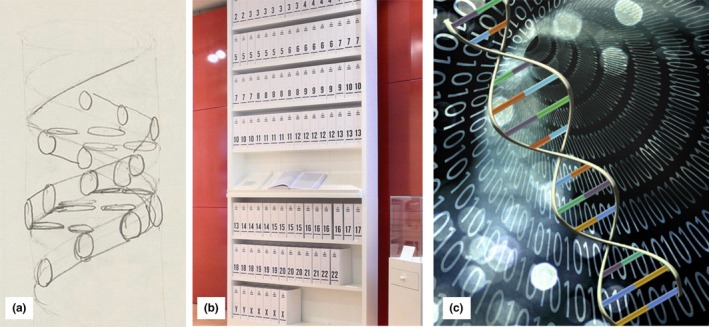
The developing aesthetic of genomics. (a) A pencil sketch of the structure of DNA by Francis Crick (Wellcome Images, 2014). (b) A printout of the human genome in a series of 118 books at the Wellcome Collection, London (Russ London, 2010). (c) A visualisation representing the power of bioinformatics software for analysing genetic data which combines the double helix, binary code and a singularity (Phys.org, 2014).

At this latter extreme, the aesthetic of genomics is an important part of the “sequencing singularity” vision. Previous work has explored the gene as a “cultural icon” beyond its strict biological definition (Nelkin & Lindee, 2004), and Franklin (2000, p. 223) memorably unpacked the genetic imaginary using *Jurassic Park,* showing “the means by which nature is remade as technique.” The aesthetic of genomics has changed over time, but a central theme has always been an emphasis on the tiny physical size of DNA in relation to its immense significance. First the double‐helix strand of DNA; then reams of text representing the “decoded” human genome; and most recently visualisations linking genomics to computing (Figure 2) all represent genomics as dealing simultaneously with the tiny, complex and powerful. It is this power which the “sequencing singularity” vision promotes in response to the international threat visualised on nextstrain.

The contemporary visual culture of EID surveillance and in turn the implicit geography of the “sequencing singularity” are dominated by two extremes: at one end of the scale, global mapping gives a sense of vulnerability to dangerous outbreaks; at the other end of the scale, sequenced DNA gives a sense of powerful knowledge of the biology of the pathogen. Other scales are conspicuous by their absence. We argue that expanded use of portable sequencing will both enable and necessitate thinking about EIDs and genomic data at scales other than the international and the genomic. In contrast to the smooth workings of EID surveillance in Gardy & Loman's imagined scenario, unexpected problems and opportunities will arise if ubiquitous portable sequencing facilitates the integration of genomic data into a global EID surveillance system. Just as EIDs present a fundamentally unpredictable problem to public health, unexpected states of affairs could arise from the widespread use of portable sequencers. We suggest, therefore, that the sequencing singularity vision should be considered with cautious scepticism for two reasons: it does not and cannot account for all significant consequences of widespread portable sequencing for EID surveillance, and – perhaps more significantly – the vision's attendant geography forecloses emergent opportunities. Of course, it would be contradictory for us to suggest that we ourselves can predict the consequences of ubiquitous sequencing. Nevertheless, we use two possible examples to demonstrate the shortcomings of the sequencing singularity vision and to emphasise a need for researchers to challenge implicit assumptions, move beyond taken‐for‐granted states of affairs, and be open to new and different topologies of EID research. We now discuss two illustrative examples, beginning with the question of biological sovereignty.

## 4 RESCALING BIOLOGICAL SOVEREIGNTY

Biological sovereignty is the assertion of ownership and use rights over biological samples and the data generated from them (Hinterberger & Porter, 2015), and has been a central issue in debates about EID surveillance. Typically, disputes have occurred between large global institutions and nations, but here we argue that portable sequencers could make individuals – at first researchers and early adopters using the technology for specific EID surveillance, but increasingly others, including members of the public – the gatekeepers of sequencing data and therefore necessitate their increased involvement in these disputes. Of course, “individuals” are already deeply involved with these disputes – for example, through their participation in democratic life, their scientific research, or their own health – and we do not wish to suggest otherwise. Involvement via portable sequencers is also dependent on both the device and the infrastructure supporting it (a point we return to at the end of this section), so it is important to continue to consider the whole system. We do not address this complexity in great depth: our argument is simply that portable sequencers shift the scale of agency and control, and that this may necessitate new and different ethical and political approaches.

In the face of the transport of biological data and the “dissolution of the bounded, autonomous organism,” nations have clashed in their attempts to claim sovereignty over “partible bits of biological life,” including viral genomes (Hinterberger & Porter, 2015, p. 363). For example, in 2012 a Saudi Arabian virologist cultured an unidentified virus from a patient, and sent the sample to the Erasmus Medical Center (EMC) in the Netherlands to be sequenced. Dutch scientists sequenced the sample and identified it as a novel coronavirus: Middle East respiratory syndrome‐related coronavirus (MERS‐CoV). EMC then patented the sequence of the viral genome before the Saudi Arabian government were aware of the isolate – a “normal thing to do” in the words of one virologist (Mayer, 2013), and a reasonable “defensive strategy” according to another, who notes that patenting assists in asserting rights over future work such as diagnostic tests (Butler, 2013). These opinions were not shared by the Saudi Arabian government, leading to an argument over whether national procedures for reporting potential novel pathogens had been followed and prompting debate about the ethics of intellectual property surrounding pathogen genome sequences (Butler, 2013). The dispute was followed by the termination of the employment of the virologist who had mailed the sample (MacKenzie, 2012). The Saudi Arabian government enacted its own biological sovereignty over the isolate, and claimed that the Dutch patent had impeded its own efforts to track the virus (MacKenzie, 2013). Such disputes are often characterised as being solely about national–international tensions, but Stephenson (2011) has convincingly argued that biological sovereignty over EIDs actually involves rival global health security aggregates. Viewed in this way, Indonesia's withdrawal from WHO's virus‐sharing scheme in 2007 after Indonesian samples were transferred for use by a pharmaceutical company without consultation was not simply a biological sovereignty dispute where a nation state refused to cooperate with an international health organisation, but a complex debate involving global health players such as “pharmaceutical companies, other international agencies such as the World Bank and the IMF, private global funds …, NGOs, and many different government departments and units” (Stephenson, 2011, p. 626).

Hinterberger and Porter (2015) argue that biological sovereignty claims gain purchase from their “tethering potential” (p. 362). That is, the strength of the claim depends upon the certainty with which a sample (and the sequences produced from it) can be anchored in place and time to a point of origin. The spur to establishing this anchor often comes from nations with less authority in global biomedicine who are disadvantaged by superficially straightforward and unobjectionable “paradigms of open science” (Hinterberger & Porter, 2015, p. 362). Previous work on the emerging forms of biological sovereignty in the context of EIDs has focused at the level of the national–international disputes that have been created by the frequent asymmetries in power produced when samples from less wealthy countries have sequencing data produced for them in wealthier countries. Although these asymmetries are not always simplistically linked to national wealth – for example, in the case of MERS‐CoV, Saudia Arabia and the Netherlands have similar GDPs (World Bank, 2017) – the general concentration of sequencers in wealthier countries and of pathogens in less wealthy countries and the consequent movement of samples between nations has meant that questions of biological sovereignty have historically been posed and considered at the international scale. Discussion has been about how countries and other international aggregates exert sovereignty over pathogen genomes, illustrated by dramatic case studies from outbreaks. The international scale also dominates the implicit geography of the “sequencing singularity” vision, which assumes that cheap portable sequencers have the potential to prevent such disputes by eliminating the asymmetry that underlies them: if the ability to sequence pathogens is distributed more equitably worldwide, more pathogens will be sequenced in situ, and establishing sovereignty over samples and the data derived from them will be straightforward.

It may be that the advent of portable sequencing could reduce these kinds of international dispute. However, the international scale – which both dominates and is reinforced by the “sequencing singularity” vision – may not be the only one on which we need to think about biological sovereignty, and we must be wary of allowing its prominence to occlude new ethical and political questions. In particular, the idea of a “portable DNA sequencer coupled to a smartphone” (Gardy & Loman, 2018, p. 16) suggests that the *individual person* – notably absent from current discussions of data sharing in EIDs – could become the relevant unit of reference in disputes. We have already seen above that international disputes can arise from individual actions and have personal consequences (such as losing a job), but could individuals themselves claim ownership over samples and data? Portable sequencers may liberate genomic data from one, static, laboratory and its norms and values, eliding the movement that causes international disputes, but their use in the field both requires and constitutes the creation of another (mobile) “laboratory” – one whose governing norms and values have yet to be established. The problem of biological sovereignty may be reduced at the international scale only to emerge at an interpersonal one: responses would need to be rescaled accordingly.

We have seen above that EID researchers considering these ethical dilemmas at an international scale often appeal to mutual obligations between nations for their citizens. If these dilemmas emerge at an interpersonal scale, their resolution may require new orderings of values. Data governance norms developed in a time when sequencers were expensive and stationary cannot necessarily be expected to hold once sequencers can be carried around in a pocket. After the Indonesian government announced their withdrawal from WHO's virus‐sharing scheme, Holbrooke and Garrett (2008) argued that Indonesia had a powerful moral obligation to share influenza samples with WHO because “failure to make viral samples open‐source risks the emergence of a new strain.” Stephenson (2011) describes this argument as invoking “a fantasy of data sharing” (p. 625). Extending this fantasy to the level of individual – as the sequencing singularity does – raises the question of whether individuals are morally obligated to make their samples open‐source for the same reasons. The unpredictable and potentially apocalyptic consequences of “emergence” imply that a single individual refusing to share their data could significantly delay the response to an outbreak. In a world where portable sequencers could be carried around, would we all be morally obligated to continuously upload information from them? Furthermore, many people might feel additionally uncomfortable if corporations were involved in the data processing, upload and storage – all of which seems highly likely if current trends continue. For example, one can easily imagine that users might have to forfeit rights to their own data as a condition of free sequencing at the point of use. These questions can be extended beyond genomic data. In the face of the risk of “emergence of a new strain” do we all have a moral obligation to share any and all relevant data (the restaurants we eat at, the houses we visit, the people we interact with, etc.) with governments and international agencies to aid their (EID) surveillance efforts?

There is some precedent to draw on if portable sequencing becomes both individualised and ubiquitous as a mobile technology. If genomic data becomes yet another “digital information stream” to be integrated with others already in existence, one way to develop the ethical topography of the sequencing singularity could be to orient it towards the kind of legal frameworks currently associated with smartphone data. Currently, individual smartphone users ostensibly control how data collected by their devices is used. In order to use smartphone software individuals must consent, with the push of a button (or, rather, the tap of a screen), to end‐user license agreements (EULAs) specifying the limits of the acceptable use of their data by the software provider. However, users typically assent to these EULAs without significant scrutiny. For example, in 2010, 7,500 GameStation customers assented to a EULA that contained a joke clause stating that they agreed to surrender their “immortal soul” to GameStation “within 5 (five) working days of receiving written notification” (Pinsent Mason, 2010). As a model for the governance of health data, such agreements therefore likely fall short of the threshold for informed consent, which requires not only informing the person consenting about the “aims, methods, sources of funding, any possible conflicts of interest, institutional affiliations … anticipated benefits and potential risks,” but also ensuring that they have understood this information (World Medical Association, 2013, p. 2193).

That portable sequencers, as handheld personal devices linked to the Internet, might be analogous in some ways to smartphones also suggests that they could come to embody similar power dynamics. Despite perhaps naive hopes that the Internet could bring “an escape from the boundaries of race” (Daniels, 2013, p. 695), it has been widely recognised that “whiteness and maleness serve as a default identity of internet users” (Noble, 2016). The white male default has yet to be decentred from digital information architectures. We cannot do justice to the issues of race, gender, class and their intersections here, but we believe these considerations are highly relevant – indeed, crucial – to the development and use of portable sequencers: they will be made and remade in the interpersonal exchanges upon which a sequencing singularity would depend.

## 5 MORE‐THAN‐HUMAN SURVEILLANCE AND MICROBIAL NOISE

While portable sequencing might require EID researchers’ thinking on biological sovereignty to move down the scale from the international level, the same technology could facilitate another shift at the opposite end of the scale: up from the level of the genome to that of the microbiome. Portable sequencers have the potential to indiscriminately sequence all genetic material from samples without a species bias. This raises the prospect that surveillance datasets will include data not just from designated pathogens, but also from commensal microbes and even humans; sequencing a sample transforms these different forms of life into digital data that is technologically indistinguishable with respect to how it is processed and stored (if still separable by bioinformatic analysis into different species). Recent work on the human microbiome has utilised sequencing methods to investigate the ecology of the microbial communities that inhabit our bodies, with the number of bacterial cells estimated to be similar to the number of human cells (Sender et al., 2016). As Lorimer (2016) puts it: “a great deal of ‘us’ is not us” (p. 57). This increasing appreciation of the importance of human–microbe relations has prompted considerations of “microbiopolitics,” which considers “the elaboration of appropriate human behaviors vis‐a‐vis microorganisms engaged in infection, inoculation, and digestion” (Paxson, 2008, p. 17). With the recognition that microbial communities are often ecologically linked to human practices, questions of ownership have also been raised previously by anthropologists (Benezra et al., 2012). While we cannot provide a satisfactory overview of this growing body of social science literature on the human microbiome, we wish to bring some thoughts to bear on the problem we are considering. The use of portable sequencers in EID surveillance will lead to the accumulation of metagenomic data from the human microbiome, and here we build on ideas from microbiome research to consider some possible consequences.

Emerging infectious disease surveillance in the “sequencing singularity” vision recognises a particular relationship between humans and microbes: pathogenic microbes moving between human nodes in a global network. Concentrating on the detection of these transmission paths can cast humans as little more than passive detectors for monitoring the dynamic spread of pathogens. However, this is not the only possible human–microbe relationship. Indeed, the vast majority of microbes that inhabit human bodies are adapted to long‐term residence and are an ever‐present part of our bodies; in Helmreich's (2014) coinage, we are “Homo microbis.” EIDs thus represent an unusual set of human–microbe relations compared with this norm. More‐than‐human geographies (Whatmore, 2006) seek to recognise the importance of such interdependent multispecies relations, for example in an account of human health as a “more‐than‐human achievement” (Lorimer, 2017, p. 103). We note that more‐than‐human geographies will become increasingly relevant as biological life is considered from a metagenomic perspective, including within EID surveillance.

The sequencing singularity vision assumes that pathogens are the subject of EID surveillance. However, such targeting requires prior knowledge of the pathogen's genome, which is unavailable for novel EIDs. Advances in technology linked to genomics have been implicated in a general “levelling of biological differences” between organisms, “reinforced by the re‐materialisation of biological entities in the guise of machine‐readable informatic codes” (Whatmore, 2006, p. 606). While a great deal of microbiome research has used sequencing of only the 16S ribosomal RNA gene to target only bacterial taxa present in samples, increasingly it is common to perform complete metagenomic sequencing of all DNA present in a sample, which presents significantly higher resolution information but also does not (in itself) methodologically discriminate between bacterial and human DNA. As metagenomic sequencing becomes cheaper and more common, surveillance will come to entail sifting through all the “levelled” mixed metagenomic data that can be isolated from a sample to find that, for example, “a novel coronavirus makes up the bulk of the microbial nucleic acid fraction” (Gardy & Loman, 2018, p. 16). The possibility of metagenomic sequencing being used in an EID surveillance network therefore raises the question of what – or who – is actually under surveillance. We suggest that the term “more‐than‐human surveillance” may be appropriate for describing this state of affairs.

On a practical level, while laboratory protocols for depleting human genetic material in samples prior to sequencing *do* exist, they are unlikely to be universally implemented or uniformly adhered to. Furthermore, they usually only succeed in reducing the amount of human DNA sequenced rather than eliminating it entirely (Feehery et al., 2013; Hasan et al., 2016). Human genomic data seems to be viewed as a “by‐product” by advocates of the sequencing singularity. For clinical researchers aiming to detect pathogens with metagenomic sequencing directly from samples, any human DNA from the patient represents “contamination” and these sequences are filtered out of downstream analysis. Other contexts provide examples where this DNA has proved to be both a valuable resource and an ethical liability. A recent study used metagenomic sequencing of saliva samples to simultaneously characterise the microbiome, detect specific pathogens, and analyse human population genetics – all from the same original data files (Lassalle et al., 2018). From a medical ethics standpoint, the procedures for managing sequencing data files should assume that human DNA may be present, because this “contamination” could both be used fruitfully in another analysis *and* could pose risks (see below). Defying the geography implicitly prescribed by the pathogen‐gentric “sequencing singularity” enables a different understanding of the indiscriminate metagenomic data generated by portable sequencers, recasting “noise” as an opportunity rather than an obstacle, albeit an opportunity raising new ethical problems. In particular, this “noise” contains information on an individual's *dynamic identity*.

An individual's genome is a static code, unchanging over their lifetime, tied to their identity at birth. The microbiome is also personal – gut microbiome samples can be used to uniquely identify individuals from groups of hundreds of others (Franzosa et al., 2015) – but generally this identifiability decreases with time because the human microbiome is a changing ecosystem that varies with external factors. For example, individuals who live together share more similar microbiomes (Shaw et al., 2017; Song et al., 2013) and events like moving abroad or diarrheal illness alter the microbiome (David et al., 2014). The microbiome therefore contains implicit information about not only individuals’ lifestyles (e.g., their diet and drug intake) but, crucially, social relationships *between* individuals (e.g., shared households and interaction patterns) (Rothschild et al., 2018). While a small subset of this information is relevant to reconstructing pathogen transmission chains in EID surveillance, the majority is unrelated to EIDs. Nevertheless, in the “sequencing singularity” this information would be available.

The fact that levelled metagenomic data encompasses information *beyond* the individual suggests a comparison with metadata on social relationships, the (in)appropriate use of which was a key factor in perhaps the most high‐profile personal data scandal of recent years involving Facebook and Cambridge Analytica (Cadwalladr & Graham‐Harrison, 2018). Metagenomic data will have (unpredictable) value above and beyond the surveillance of EIDs. The wider possibilities for this data are absent from the “sequencing singularity” vision, perhaps because they are irrelevant to its central goal. The collection and instant global sharing of such information offers opportunities for exploitation as well as for the protection of health. To view the data as a “by‐product” is to neglect these opportunities – opportunities which are unlikely to be neglected by corporate entities as they arise. Indeed, at the other extreme to enthusiasm for data sharing, Mirowski has argued that “open data” and “open science” are part of an agenda to “re‐engineer science along the lines of platform capitalism” (Mirowski, 2018, p. 171). We therefore feel that EID researchers should make time for serious consideration of the possible roles of commercial organisations when calling for a dramatic expansion of sequencing and a “singularity” that deliberately brings data streams together. We also think that a further risk is the inadequate acknowledgment that current global digital information networks rely on commercial organisations and (often) proprietary software. The ethical implications of this state of affairs have not been taken into account by the “sequencing singularity” vision – unsurprisingly, as the vision has developed in a context where academic norms around “open science” are dominant (e.g., Bedford et al., 2018; Gardy et al., 2015; Open Science Prize, 2017; Schatz & Phillippy, 2012; Zibra Project, 2016) – but we believe it is crucial to bear in mind.

To summarise, a likely consequence of widespread portable sequencers will be the production of vast amounts of sequencing data from human microbiomes. The majority of this data will be irrelevant to EID surveillance, which can be summarised as aiming to distinguish pathogenic signal from “background microbial noise” (Gardy & Loman, 2018, p. 14). However, data collected from “more‐than‐human surveillance” has other possible uses beyond EID surveillance, as it contains information not just about individuals’ lifestyles and dynamic identities, but the lives of others. Far from being extraneous, we think that when released from the “sequencing singularity” vision's restrictive topology, this “background microbial noise” could make visible more‐than‐human geographies, leading to new risks and opportunities for individuals, researchers, and government and corporate organisations.

## 6 CONCLUSION

In this article, we have argued that the “sequencing singularity” vision for the future of EID surveillance has an attendant geography drawn from the strategy and aesthetics of contemporary EID and genomic research. This geography privileges the genomic and the global at the expense of intermediate scalar points. Through discussion of some of the problems and opportunities that could arise from widespread portable sequencing – a crucial enabling technology for the “sequencing singularity” vision – we have attempted to disrupt this geography, showing that portable sequencers could both require and enable different topologies of EID research, turning researchers’ attention to questions emerging at scales currently occluded by the dominant logics of EID surveillance. We have posited that the question of biological sovereignty will render interpersonal exchanges of data more relevant, while indiscriminate genome sequencing will offer greater prominence to more‐than‐human geographies.

We have drawn on previous work considering EID data disputes about biological sovereignty to suggest that the interpersonal scale may become more significant for data disputes once the ability to sequence is in the hands of individual persons. We argue that established ethical imperatives to share data at international scales are unlikely to hold at interpersonal scales. We therefore believe that new forms of biological sovereignty are likely to emerge at the level of the individual, and that these may be enmeshed with commercial organisations, government departments, and other organisations in complex ways. The unpredictable nature of EIDs makes them a special case, and further thinking about the rights and obligations underpinning the “sequencing singularity” is required.

We have also explored the consequences of indiscriminate metagenomic sequencing without a species bias. A value judgment is involved in dismissing most of this data as “background microbial noise” (Gardy & Loman, 2018, p. 14), extraneous data within which signals of pathogen “emergence” are believed to be “hidden.” Other judgments may confer different values on this data. To return to our example of the Internet discussion system Usenet: the values associated with a technology by its early adopters are not inherent or durable properties of the technology itself. Data produced via portable sequencing for the purpose of EID surveillance could have radically different uses. It could represent not only commercial opportunity for corporations, but – when considered from a more‐than‐human perspective – opportunities to engage with the rich and complex relationships between humans and microorganisms. These relationships are part of a “folded life” in which humans actively work with complex microbial environments, rather than striving to create biosecure settings that keep disease out (Hinchliffe & Ward, 2014, pp. 142–143). Future work considering the implications of ubiquitous real‐time sequencing for these geographies of folded life will expand our understanding of these relationships.

These are just two examples; we believe that there are myriad ways in which widespread portable sequencing for global EID surveillance could and will be associated with new orderings of people, things and values. The consequences of portable sequencing technology may in many ways be as unpredictable as the EIDs its proponents seek to identify. We can only advise that, although the “sequencing singularity” vision is a powerful and in many ways attractive one, there is a need on the one hand for vigilance in our awareness that there would be consequences that it does not and cannot foresee; and on the other hand, for an alertness to emergent opportunities that could be hidden or foreclosed by its assumptions.

## AUTHORS’ NOTE

We would like to acknowledge some events that occurred during the conception and writing of this paper that played a role in shaping our thoughts and feelings. In early 2018 we came across Ruffino's (2017) article “Archaeology for Cyborgs” which addressed the question: “how do we draw the line between legitimate research and an invasion of privacy?” and stimulated our initial discussions about data sharing. Our concerns about data and personal privacy were felt all the more keenly in April 2018, when N.S. received a notification on facebook.com informing her that because one of her friends had logged into the app “This is Your Digital Life,” her own information including public profile, page likes, date of birth, and current city were “probably” shared with the app and in turn with (now‐defunct) political consulting firm Cambridge Analytica Ltd (Facebook, 2018: personal communication). Our thinking was also influenced by a widely reported case where an inadvertent surveillance system unexpectedly made visible a highly sensitive geography: the smartphone app Strava, used by many individuals to track their jogging routes and times using their GPS location, released a global heatmap of all public user activity online in late 2017. Operational security researchers noted that this map could be used to identify the secret locations of US military bases in Syria because Strava had recorded the regular jogs of military personnel around the bases (Hern, 2018). All individuals involved had freely consented (knowingly or unknowingly) to the use of their data in this way. This incident demonstrated that it is not only individual‐level privacy that is at stake when individuals consent to submit data to an aggregated global system. Finally, our sense of the difficulty of responding to the chaotic and the unpredictable was heightened by N.S.'s proximity to the Saddleworth Moor wildfire which broke out on Sunday 24 June 2018 and was still smouldering as we finalised this paper.

## END NOTES

1 There is an ambiguity in the term “data sharing” as to *what* “data” is being shared with *whom*. In the case at hand we are specifically concerned with the integration of genomic data (that is, the sharing of the As, Ts, Cs and Gs produced by sequencers) with other kinds of data. We discuss more on the *what* and *whom* in the rest of our article. (Note, in line with many in the open data movement, we use “data” as singular.)

2 Leonelli (2016, p. 70) has argued that data is “defined by the *evidential value* ascribed to [it] at specific moments of inquiry” (original emphasis). The point is instructive in this instance, where what could feasibly count as “data” for Gardy and Loman's surveillance system, with its shifting foci of inquiry, is necessarily wide ranging, incorporating any and all information available (see their scenario, discussed in Section 2).

3 In the early years of the internet, the month of September witnessed a regular influx of new users as students began college and acquired their first online accounts (Raymond, 2003).
